# Supplementary material for: Transplanted Adult Neural Stem Cells Express Sonic Hedgehog In Vivo and Suppress White Matter Neuroinflammation after Experimental Traumatic Brain Injury
Source: Stem Cells Int. 2017 Aug 30;2017:9342534. doi: 10.1155/2017/9342534 (PMC5610817; doi:10.1155/2017/9342534)
Supplement: Supplementary file 1 — Figure S1. Demonstration of in vitro neurosphere formation and multipotentiality of NSCs derived from the subventricular zone (SVZ) of adult mice. Figure S2. Immunohistochemical identification of Shh-Tom cells as neurons. Figure S3. Induced genetic in vivo labeling of neurons synthesizing Shh that project axons in the corpus callosum and cingulum. Figure S4. Distribution of Shh responsive cells in regions adjacent to the lateral ventricles. [file 9342534.f1.pdf]

Supplemental Materials

Figure S1. Demonstration of *in vitro* neurosphere formation and multipotentiality of NSCs derived from the subventricular zone (SVZ) of adult mice. A-C: Adult NSCs form neurospheres in an *in vitro* suspension culture. Phase contrast image of neurospheres with varying diameters at 3 days after isolation from the SVZ of an adult mouse (A) with higher magnification of middle set (B). NSCs isolated from the SVZ of adult C57BL/6-Tg(UBC-GFP)30Scha/J mice, shown 3 days after passage 5, form neurospheres *in vitro* (C; phase contrast) and express green fluorescent protein (C'; GFP; C"; merge) **D-E:** *In vitro* differentiation shows multipotency of adult NSCs based on immunolabeling for cell type-specific lineage markers of astrocytes (GFAP; D), neurons (MAP-2; E), and oligodendrocytes (O4; F). Scale bars = 100 mm (A, B, C-C"), 40 μm (D), 20 μm (F).

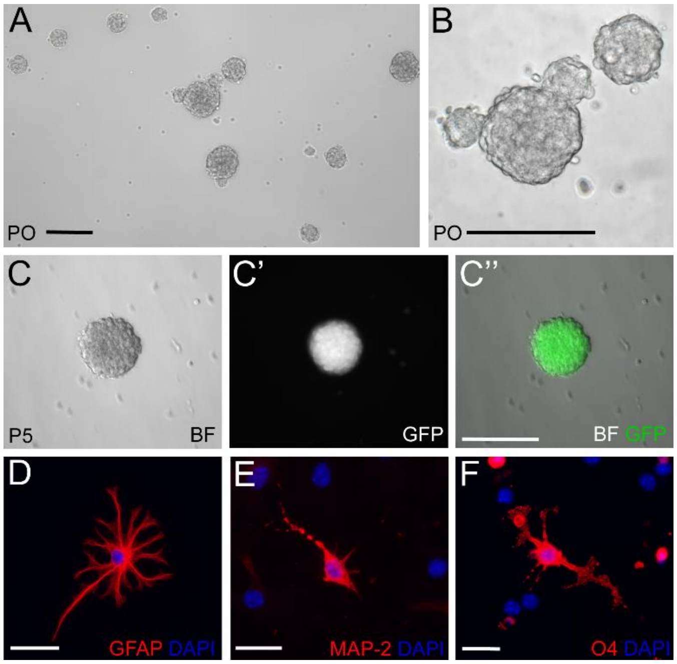

Figure S1

Table S1. Mouse lines.

	Full name	Source	RRID
Shh^{CreERT2}	*B6.129S6-Shh*$^{tm2(cre/ERT2)Cjt}$/J	Jackson Laboratories Cat #005623	IMSR:JAX_005623
Gli1^{CreERT2}	*Gli1*$^{tm3(cre/ESR1)Alj}$*/J*	Jackson Laboratories Cat #007913	IMSR: JAX_007913
R26YFP	*B6.129X1-Gt(ROSA)26Sor*$^{tm1(EYFP)Cos}$*/J*	Jackson Laboratories Cat #006148	IMSR:JAX_006148
R26tdTomato	*B6.Cg-Gt(ROSA)26*$^{Sortm14(CAG-tdTomato)Hze/}$*J*	Jackson Laboratories Cat #007908	IMSR:JAX_007908
R26mT/mG	*B6.129(Cg)-Gt(ROSA)26Sor*$^{tm4(ACTB-tdTomato,-EGFP)Luo}$*/J*	Jackson Laboratories Cat #007676	IMSR:Jax_007676
	C57BL/6-Tg(UBC-GFP)30Scha/J	Jackson Laboratories Cat #004353	IMSR: Jax_004353

Figure S2. Immunohistochemical identification of Shh-Tom cells as neurons. Coronal sections showing the cerebral cortex (A) and striatum (B) regions from *ShhCreERT2;R26tdTomato* mice. Neurons were identified by expression of the neuronal cell type-specific marker NeuN. Shh-Tom labeled cells (red) exhibit co-labeling with NeuN (green), with the overlap appearing yellow/orange (arrows). Shh-Tom neurons are distributed among cortical layers and are found near the lateral ventricle (LV). Scale bars 200 μm (A), 100 μm (B).

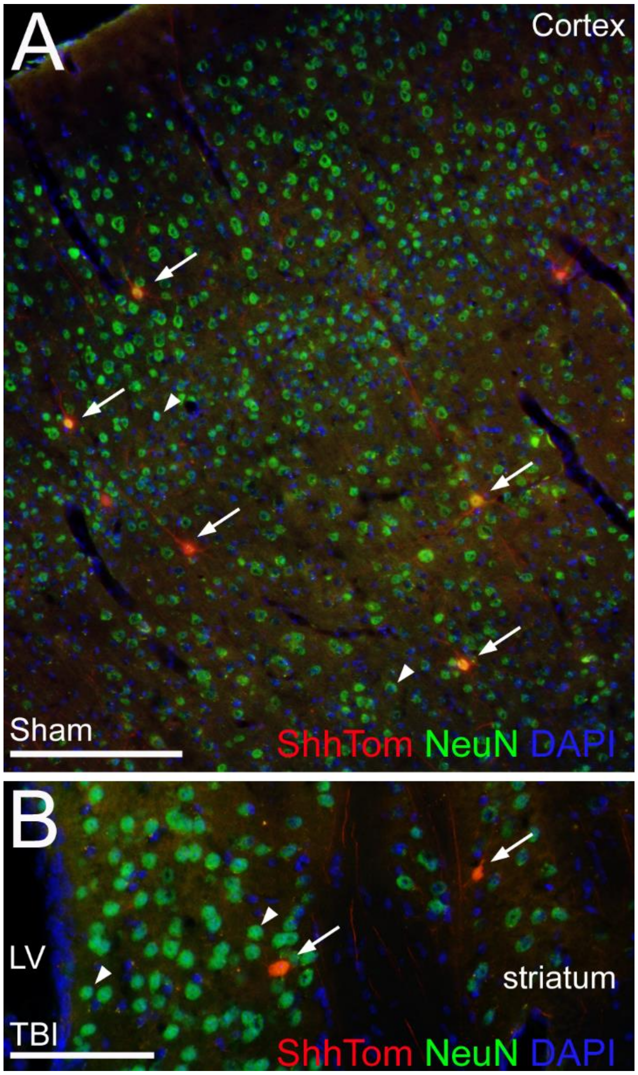

Figure S2

Figure S3. Induced genetic *in vivo* labeling of neurons synthesizing *Shh* that project axons in the corpus callosum and cingulum. Tissues were prepared as in Figure 3, which shows higher magnification examples of transplanted NSC-GFP. **A-C:** Coronal sections showing Shh-Tom labeling (red) with DAPI nuclear stain (blue) in the corpus callosum (cc) and cingulum (cg), regions, which exhibit axon damage and neuroinflammation in this TBI model. The pattern of heritable Shh-Tom labeling of axons in the cingulum and corpus callosum was similar between sham mice (A), TBI mice (B), and TBI mice that received NSC-GFP cells transplanted (C) into the lateral ventricle (LV). **D-F:** Higher magnification of Shh-Tom labeled axons in the cingulum (D) and corpus callosum (E-F). Shh-Tom axons can exhibit greater variation in diameter after TBI (E, arrow) than Shh-Tom axons in sham mice (F). Scale bar = 100 µm (A), 50 µm (D), 25 µm (F). Representative images are shown from analysis of a cohort of sham + NSC-GFP (n = 2), TBI + vehicle (n = 3), and TBI+ NSC-GFP (n = 3) mice.

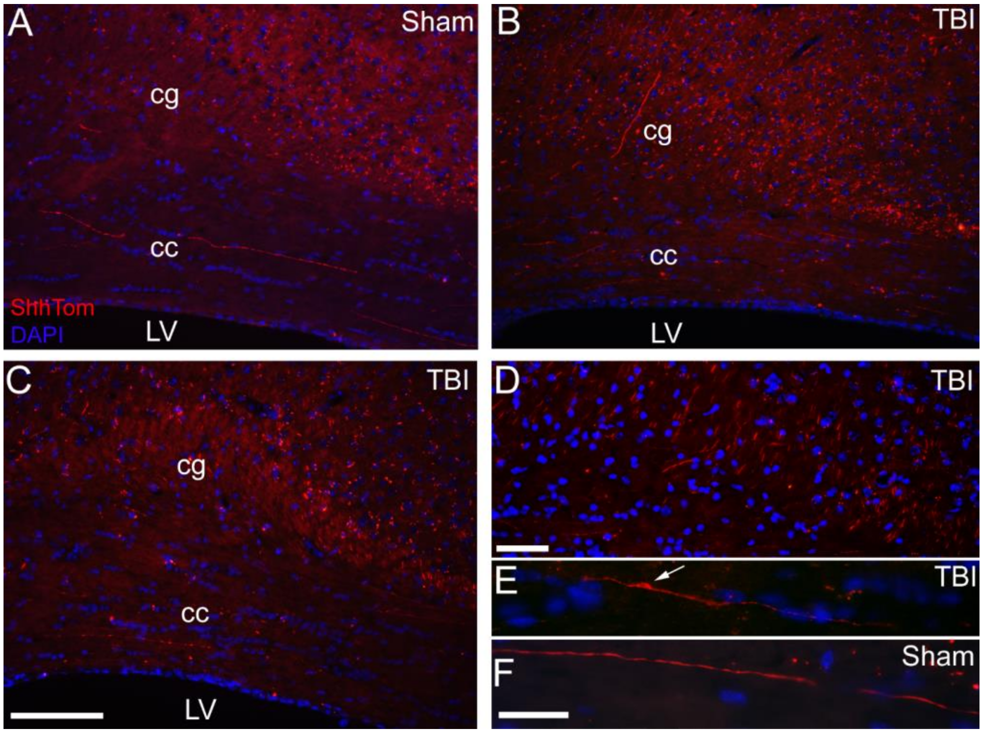

Figure S3

Figure S4. Distribution of Shh responsive cells in regions adjacent to the lateral ventricles. Tissues were prepared as in Figure 5. Coronal sections show Gli1-Tom labeling in endogenous cells of the subventricular zone (SVZ) and regions adjacent to the lateral ventricles (LV). **A-C:** *Glil1CreERT*^{2}*;R26tdTomato* mice with vehicle injections show Gli1-Tom genetic labeling of cells in the SVZ, septum, and striatum. **D-F:** *Gli1CreERT*^{2}*;R26tdTomato* mice with transplanted NSC-GFP cells (arrows), identified by constitutive green fluorescent protein, have similar Gli1-Tom expression to vehicle controls. Gli1-Tom labeled cells are not evident in the corpus callosum (cc) regardless of injury or NSC transplantation conditions. Representative images from cohorts of naïve + vehicle (n = 3), naïve + NSC-GFP (n = 2), sham + vehicle (n = 4), sham + NSC-GFP (n = 4), TBI + vehicle (n = 3), and TBI + NSC-GFP (n = 4).

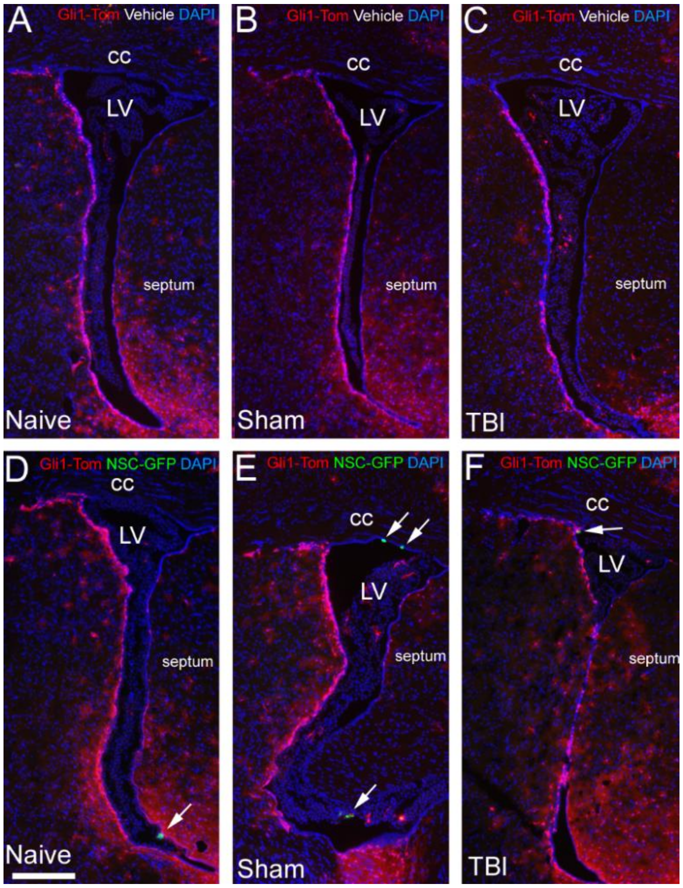

Figure S4
